# Reducing the internal reorganization energy *via* symmetry controlled π-electron delocalization†

**DOI:** 10.1039/d2sc01851a

**Published:** 2022-05-24

**Authors:** Chi-Chi Wu, Elise Y. Li, Pi-Tai Chou

**Affiliations:** Department of Chemistry, National Taiwan Normal University No. 88, Section 4, Tingchow Road Taipei 116 Taiwan eliseytli@ntnu.edu.tw; Department of Chemistry, National Taiwan University No. 1, Section 4, Roosevelt Road Taipei 106 Taiwan chop@ntu.edu.tw

## Abstract

The magnitude of the reorganization energy is closely related to the nonradiative relaxation rate, which affects the photoemission quantum efficiency, particularly for the emission with a lower energy gap toward the near IR (NIR) region. In this study, we explore the relationship between the reorganization energy and the molecular geometry, and hence the transition density by computational methods using two popular models of NIR luminescent materials: (1) linearly conjugated cyanine dyes and (2) electron donor–acceptor (D–A) composites with various degrees of charge transfer (CT) character. We find that in some cases, reorganization energies can be significantly reduced to 50% despite slight structural modifications. Detailed analyses indicate that the reflection symmetry plays an important role in linear cyanine systems. As for electron donor–acceptor systems, both the donor strength and the substitution position affect the relative magnitude of reorganization energies. If CT is dominant and creates large spatial separation between HOMO and LUMO density distributions, the reorganization energy is effectively increased due to the large electron density variation between S_0_ and S_1_ states. Mixing a certain degree of local excitation (LE) with CT in the S_1_ state reduces the reorganization energy. The principles proposed in this study are also translated into various pathways of canonically equivalent π-conjugation resonances to represent intramolecular π-delocalization, the concept of which may be applicable, in a facile manner, to improve the emission efficiency especially in the NIR region.

## Abstract 2

The reorganization energies may be significantly reduced by molecular symmetry effect.

## Graphical abstract

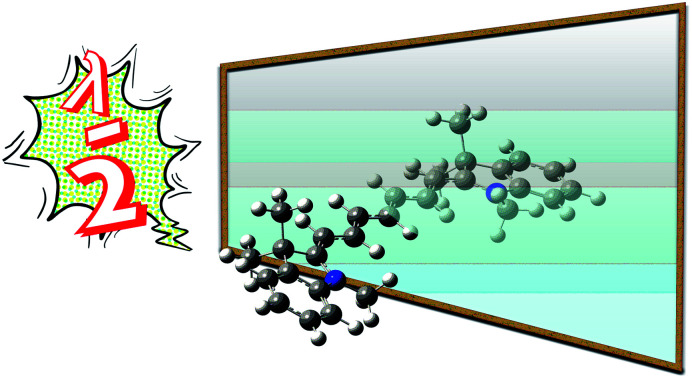

## Introduction

**Fig. 1:**
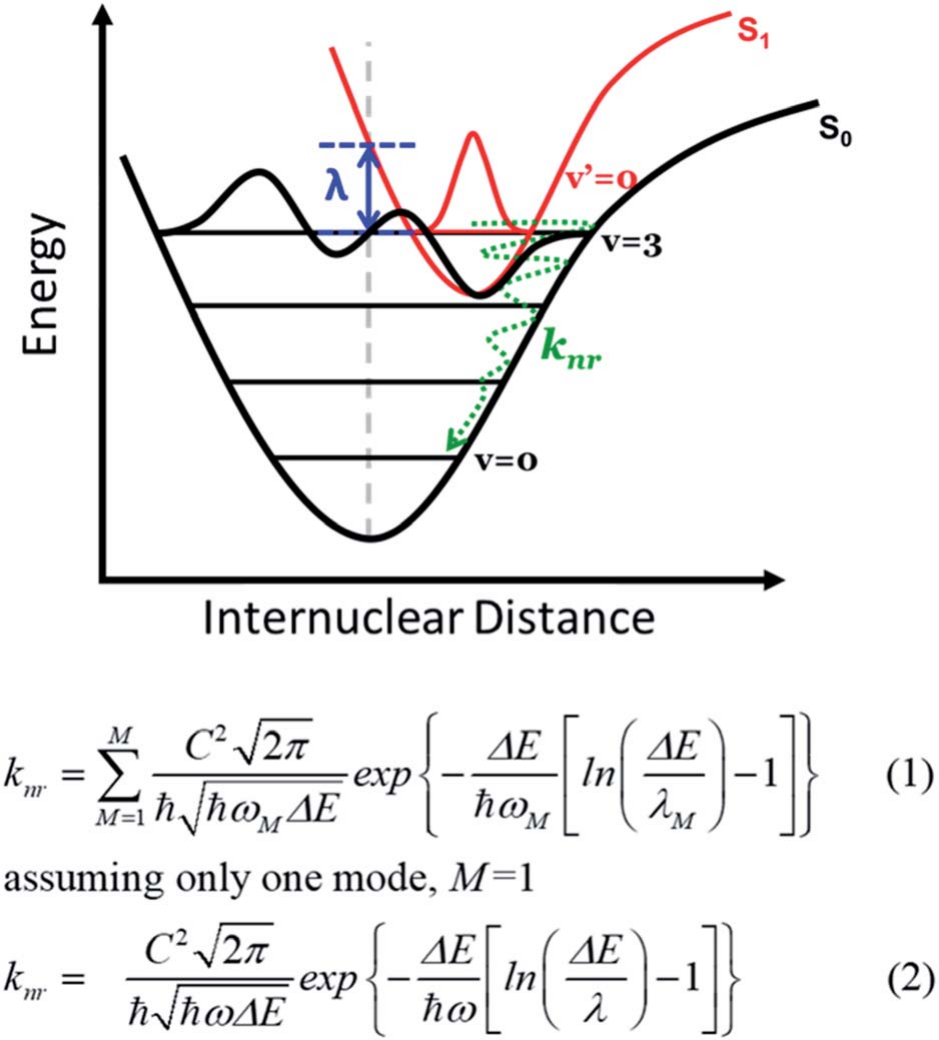
The coupling between S_1_ (at zero-point energy *v*′ = 0) and high-lying vibrational states (*v* = *m* of S_0_, *e.g. m* = 3) involved in the transition. Also shown are eqn (1) and (2) for the emission energy gap law, where *k*_nr_ is the non-radiative decay rate deduced according to the energy gap law. In eqn (1) and (2), *C* is an effective electronic coupling constant, Δ*E* is the energy gap between the two designated electronic states for the transition, *ω*_M_ is the angular frequency of the promoting vibrational modes, and *λ*_M_ is the inner-shell reorganization energy of that promoting mode. Assuming only one coupling mode, eqn (1) can be simplified to eqn (2).

Reorganization energy plays an important role in material efficacy for optoelectronic and electrical devices such as organic light emitting diodes (OLEDs)^1–4^ and organic field-effect transistors (OFET).^5–8^ Based on the semiclassical Marcus theory, a small reorganization energy facilitates acceleration of the inter-molecular charge hopping rate^9–12^ and improves the emission quantum yield for intra-molecular excitations by reducing the non-radiative decay rate (Fig. 1).^13,14^ In the field of lighting materials, strategies to reduce the reorganization energy by innovative molecular designs have been the focus of many recent studies with an aim to maximize the emission yield. In theory, the reorganization energy (*λ*) is a sum of a major inner-sphere (or internal, (*λ*_inner_) part, specified in Fig. 1), and a minor external, or outer-shell (*λ*_outer_) part.^15–17^ The inner-sphere reorganization energy mainly includes contributions from geometry relaxations in the excited state caused by charge injection or electronic transition processes.^18–23^ In practice, the inner-sphere reorganization energy may be suppressed by increasing the molecular rigidity in the solid matrix incorporating stacking and/or self-assembly,^24–28^ or by decreasing the bond length variation upon excitation *via* extending the π conjugation skeleton or enhancing the local non-bonding characteristics in organic molecules.^29–32^ Alternatively, a lower reorganization energy has also been observed in systems with higher molecular symmetry and/or more delocalized molecular orbitals. Systems such as ring-fused linear π-conjugated molecules with *D*_2h_ symmetry have been recently reported with reorganization energies under 100 meV.^33^ Nevertheless, a complete theoretical rationalization for the symmetry effect on the reorganization energy has not been proposed. The reorganization energy in the inner-shell part is especially critical for compounds emitting in the near-infrared (NIR) region, where a specific non-radiative decay pathway becomes dominant. This quenching process incorporates coupling between electronically excited states and high-lying vibrational levels of the ground state involved in the transition (see Fig. 1) with mathematical expressions written in eqn (1) and (2) for multiple and single vibrational modes, respectively.^34^ Apparently, according to eqn (2), *k*_nr_ increases as *λ*_inner_ increases and becomes significant when Δ*E* decreases; the latter specifies the origin of the emission energy gap law.^34–36^

Straightforwardly, the reorganization energy can be calculated by the total energy difference of the excited system between the vertical ground state geometry and that in the relaxed structure, as proposed in the adiabatic potential method.^36^ This, however, is computationally expensive for large molecular systems as full geometry optimizations in the excited state are requested. Alternatively, other methods such as the orbital vibronic coupling (OVC) method^37,38^ have also been proposed and utilized to estimate the reorganization energy by the phase of frontier molecular orbitals and their energy variations upon excitation. More specifically, the magnitude of reorganization energies of different electronic states can be gauged by transition densities, which presents spatial overlap of the HOMO and LUMO. For example, it is well known that simple polycyclic aromatic rings with the first excited state in the ^1^L_b_ symmetry exhibit lower reorganization energy than those in the ^1^L_a_ symmetry, based on Platt's classification.^39–41^ Similar principles have been extended and applied to other types of compounds, but the discussions are largely limited to cyclic systems.^42–48^ Empirical design principles correlating the molecular geometry and the electronic structure characteristics in terms of the reorganization energy for linear molecules or organic intramolecular charge transfer type systems are relatively scarce, especially for realistic chromophores with application potentials beyond simple model systems.

In this study, we explore the effect of molecular symmetry on the magnitude of the reorganization energy using first-principles investigations on two types of model systems: linear cyanine compounds and electron donor–acceptor (D–A) systems. These molecules contain the basic molecular motifs for potential NIR-emitting candidates^49–53^ and serve as ideal theoretical models to demonstrate the effect of molecular symmetry. Cyanine dyes consist of a conjugated polymethine bridge between two nitrogen terminals and have been extensively applied in the photographic industry and bioimaging.^53–60^ In yet another approach, donor–acceptor (D–A) templates are inspired by reported systems which have shown efficient intramolecular charge transfer and a low band gap with decent emission quantum yield.^61–65^ Prototypes are 6-TPA–NO and 6-TPA–NT, consisting of electron-withdrawing moieties, naphtho[1,2-*c*:5,6-*c*′]bis[1,2,5]oxadiazole (NO) or naphtho[1,2-*c*:5,6-*c*′]bis[1,2,5]thiadiazole (NT),^66–73^ respectively, and electron-donating groups, triphenylamines (TPAs).^74^ Whereas many factors cause deactivation besides the reorganization energy, for instance, reabsorption^75^ and rotation quenching induced by an electron-withdrawing terminal,^76^ these will not be discussed here as they are irrelevant to the transition on π delocalization (*vide infra*). In this study, various symmetric, asymmetric as well as trimeric cyanine dyes and D–A models are considered with detailed electronic structure analyses. We find that the molecular symmetry has a dominant effect on the reorganization energy, as manifested by the even–odd number of carbon atoms in the polymethine chain of the cyanines, the reflection symmetry of the molecular geometry and the substitution positions in the D–A systems. At present, the quantum yields of NIR emitters are still primarily limited by the energy gap law, and systematic improvement methods are lacking. By demonstrating the symmetry effect on these backbones of NIR systems, we anticipate that the quantum efficiency of NIR molecules can be strategically enhanced *via* structural design.

## Computational details

Density functional theory (DFT)^77,78^ calculations were carried out for structural optimization at the ωB97XD^79^/6-311+G(d,p) level and b3lyp^80^/6-311+G(d,p) level for linear cyanine models, the ωB97XD/6-31G(d) level for trimeric cyanine models, and the ωB97XD/6-311+G(d,p) level for donor–acceptor models by using the Gaussian 16 program package.^81^ The energy minima are confirmed with no imaginary frequencies by vibrational frequency calculations. Optical excitation energies and excited state geometries are obtained by time-dependent density functional theory (TDDFT) calculations.^82,83^ Natural transition orbitals (NTOs)^84^ are evaluated to characterize the nature of the first excited states for those models with complex transition compositions. Solvent effects were considered using the polarizable continuum model (PCM)^85^ with dichloromethane (dielectric constant *ε* = 8.93)^86^ and cyclohexane (*ε* = 2.02)^87^ solvents for cyanine and D–A compounds, respectively.

Internal reorganization energies (*λ*_int_), based on Marcus theory,^40,88,89^ are calculated using the following equation,

3 *λ*_int_ = *E*_S_1_@S_0__ − *E*_S_1_@S_1__

where *E*_S_1_@S_0__ and *E*_S_1_@S_1__ refer to the Franck–Condon first excited state (S_1_) energy in the ground state S_0_ optimized structure and the S_1_ optimized structure, respectively. Molecular transition density of the lowest ππ*-excited state, calculated from the diagonal term of the transition density matrix, is visualized to rationalize the relative magnitude of the reorganization energy.^39,90,91^

## Results and discussion

### Linear and 2D cyanine models

**Scheme 1:**
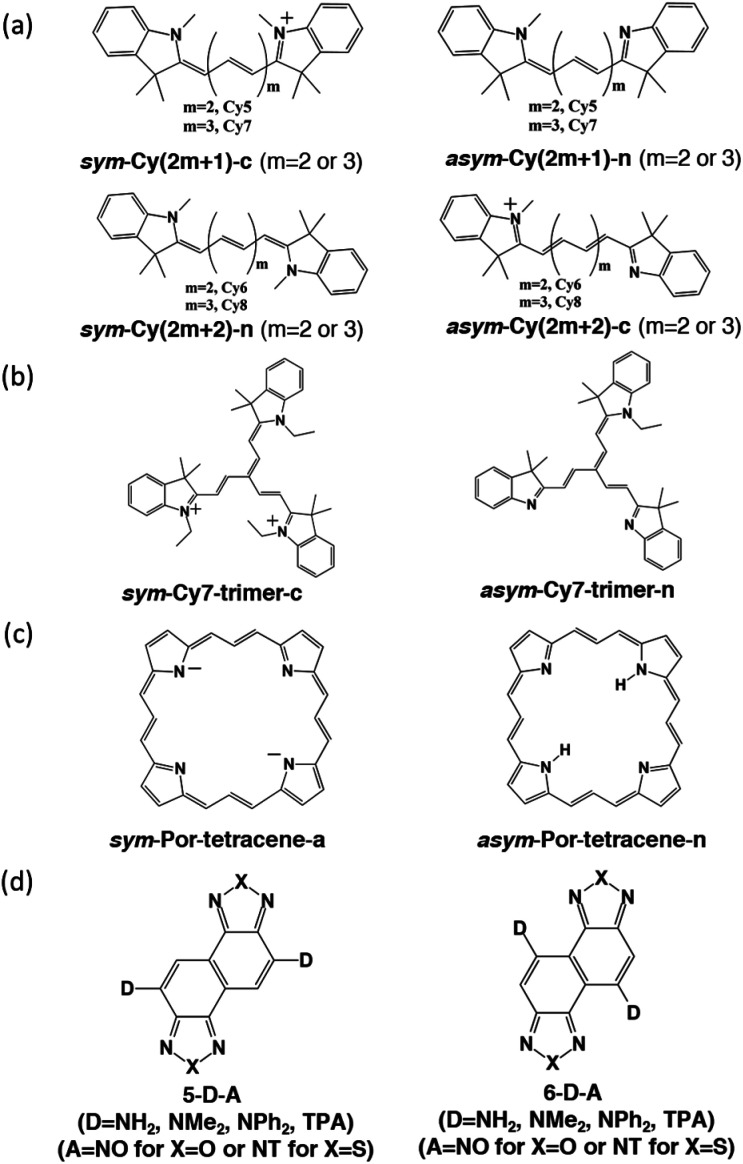
Computational model systems investigated in this study: symmetric and asymmetric (a) linear cyanines and (b) trimeric cyanines, (c) porphyrin-6,13,19,26-tetracene molecules and (d) donor–acceptor molecules with different substitution positions (5-D–A and 6-D–A).

**Fig. 2:**
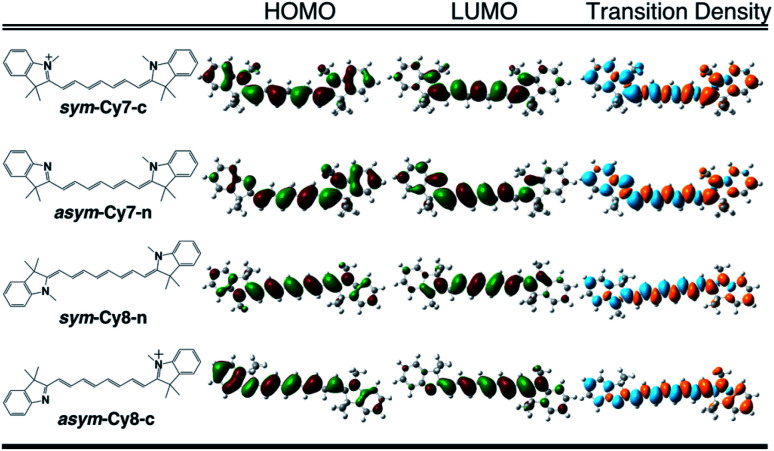
Frontier molecular orbitals and transition densities of symmetric and asymmetric cyanine systems (*m* = 3) containing an odd (Cy7) or even (Cy8) number of carbon atoms in the polyene chain.

We designed and computed various symmetric and asymmetric cyanine and donor–acceptor systems, as shown in Scheme 1. For typical cyanines containing an odd number of carbon atoms in the conjugated chain, neutral asymmetric cyanine molecules, *asym*-Cy(2*m* + 1)-n (*m* = 2 or 3), are constructed by removing one ethyl group from the nitrogen terminals on cationic symmetric cyanines, denoted as *sym*-Cy(2*m* + 1)-c. For comparison, we also consider cyanine congeners containing an even number of carbon atoms in the conjugated chain, where the symmetric and the asymmetric cyanines are neutral and cationic systems, represented as *sym*-Cy(2*m* + 2)-n and *asym*-Cy(2*m* + 1)-c, respectively. Structurally, geometrical isomers should exist for the Cy(2*m* + 1) and Cy(2*m* + 2) series; here, we adopt geometrical isomers where the two indole-like moieties are in the *cis* and *trans* configuration for Cy(2*m* + 1) and Cy(2*m* + 2), respectively, for the simplification of discussion (see, Scheme 1, *vide infra*). Calculated optical excitations and their compositions for the optimized cyanine series are listed in Tables S1 and S2.† In the cyanine systems, molecular orbital (MO) distributions of the ground S_0_ and the optimized S_1_ states are very similar, as well as the contributions of the MO transitions. For all calculated cyanine systems, clearly, the lowest lying excited state, *i.e.*, the S_1_ state, is dominated by the HOMO–LUMO transition, which corresponds primarily to the π → π* transition of the conjugated cyanine backbones, as depicted in Fig. 2, along with their respective transition densities. The more symmetric molecular orbital distribution implies a relatively higher degree of local excitation (LE) in the symmetric cyanines, *sym*-Cy(2*m* + 1)-c and *sym*-Cy(2*m* + 2)-n, with respect to a more charge-transfer (CT) type excitation from the 1-ethyl-3,3-dimethylindoline end to the 3,3-dimethylindolenine end in the asymmetric cyanines, *asym*-Cy(2*m* + 1)-n and *asym*-Cy(2*m* + 2)-c.

Table 1 presents the reorganization energies calculated using eqn (3) for the cyanine systems. Interestingly, most linear cyanine systems exhibit very similar values for reorganization energies, around 13 to 15 kcal mole^−1^, except for the symmetric cyanines with an odd number of carbon atoms, *sym*-Cy5-c and *sym*-Cy7-c, which exhibit much smaller reorganization energies at around 6 kcal mole^−1^. The cyanine systems with an odd number of carbon atoms, as presented by the shaded row in Table 1, thus give rise to a distinctively large reorganization energy ratio at around 2 for the asymmetric *versus* symmetric counterparts. It is surprising that such a slight structural modification causes a drastic reduction in the reorganization energy, and that this phenomenon occurs only for systems with an odd number of carbon atoms in the polyene chain. A careful inspection reveals different symmetry elements, *σ* or *C*_2_, for the symmetric cyanines with odd or even carbon numbers, respectively, as reflected by the *σ* or inversion symmetry found in their respective π molecular orbital distributions.

**Table 1:** Reorganization energies (kcal mole^−1^) of symmetric (*λ*_*sym*_) and asymmetric (*λ*_*asym*_) cyanine systems and their relative magnitudes (*λ*_*asym*_/*λ*_*sym*_)

	*λ*_*sym*_	*λ*_*asym*_	*λ*_*asym*_/*λ*_*sym*_
Cy5	6.44	12.41	1.93
Cy6	13.79	15.81	1.15
Cy7	5.80	13.70	2.36
Cy8	13.37	13.45	1.01
Cy7-trimer	8.44	12.73	1.51
Por-tetracene	∼0.00^a^	6.71	N/A

a A reorganization energy of −0.78 kcal mole^−1^ is calculated with wb97Xd/6-311+G**. The highly symmetric Por-tetracene systems give rise to degenerate molecular orbitals and virtually identical S_0_*versus* S_1_ structures.

**Fig. 3:**
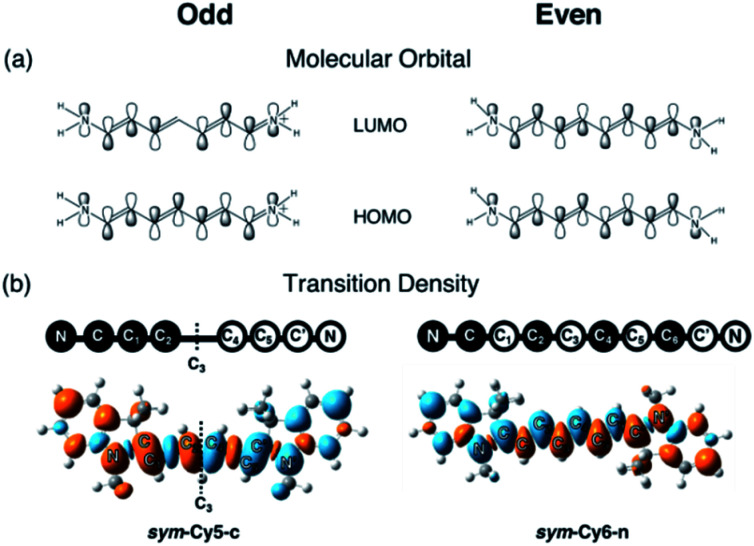
(a) Sketches of frontier molecular orbitals and transition densities for polyenes with an odd or even number of carbon atoms in the conjugated backbones. (b) The calculated transition densities of *sym*-Cy5-c and *sym*-Cy6-n for comparison. The dashed line represents the nodal plane across C_3_.

The same symmetry element can be identified in the transition densities of linear cyanines. More specifically, the transition densities of the symmetric odd-number systems, *sym*-Cy(2*m* + 1)-c, present the *σ* symmetry with a node across the central carbon atom, as shown by *sym*-Cy7-c in Fig. 3. This phenomenon can be more clearly understood by the corresponding sketches shown in Fig. 3, where the transition densities of diaminopolyenes containing odd and even numbers of carbon atoms in the backbone are constructed, in a qualitative manner, using the frontier molecular orbital distribution neglecting individual atomic orbital coefficients. As a result, for each atom site, the p_*z*_ orbital in the same phase in the HOMO and LUMO gives rise to a positive sign in the transition density, represented by the black circle, whereas the p_*z*_ orbital in the opposite phase in the HOMO and LUMO gives rise to a negative value represented by the white circle. A nodal plane across the central carbon atom naturally occurs in the transition density due to the *σ* symmetry in the odd type polyene corresponding to *sym*-Cy7-c. This excitation is classified as ^1^B_b_ in Platt's notation, analogous to the ^1^L_b_ symmetry for cyclic systems. For the *sym*-Cy8-n representing even-type polyene systems, however, the transition density shows a high extent of alternating signs with multiple nodes across the C–C bonds. This is characteristic of a bond-weakening excitation from a bonding to an anti-bonding orbital that leads to a larger reorganization energy, as can be estimated by the energy gap coupling constants in the orbital vibronic coupling (OVC) method.^37,90^

We also designed and investigated a series of fused trimer-like structures with symmetric or asymmetric branches as well as porphyrin-like structures, represented as *sym*/*asym*-Cy7-trimer and *sym*/*asym*-Por-tetracene. The corresponding structures are depicted in Scheme 1. In fused symmetric and asymmetric Cy7 trimers, a similar trend can be observed. The symmetric trimer with three equivalent ethyl-substituted branches shows a 1.5 times reduction in the reorganization energy compared to the asymmetric trimer that possesses one ethyl-substituted and two unsubstituted branches. It appears that an extension from the linear to the trimeric structure does not guarantee a more striking symmetry effect as the charge transfer excitation becomes less directional in a tripod system, as shown in Fig. S2.† On the other hand, unlike the more or less planar linear cyanines, the three branches of the optimized trimeric systems slightly rotate away from planarity like blades of a fan, which may have contributed other unexpected factors. In porphyrin-like, planar structures, the reorganization energy is expected to be lower than that of the linear systems. In asymmetric porphyrin-6,13,19,26-tetracene, *asym*-Por-tetracene, the reorganization energy is about 6.71 kcal mole^−1^, and in the symmetric structure, *sym*-Por-tetracene, due to its local excitation character the reorganization energy vanishes (Fig. S3†).

### Donor–acceptor models

**Fig. 4:**
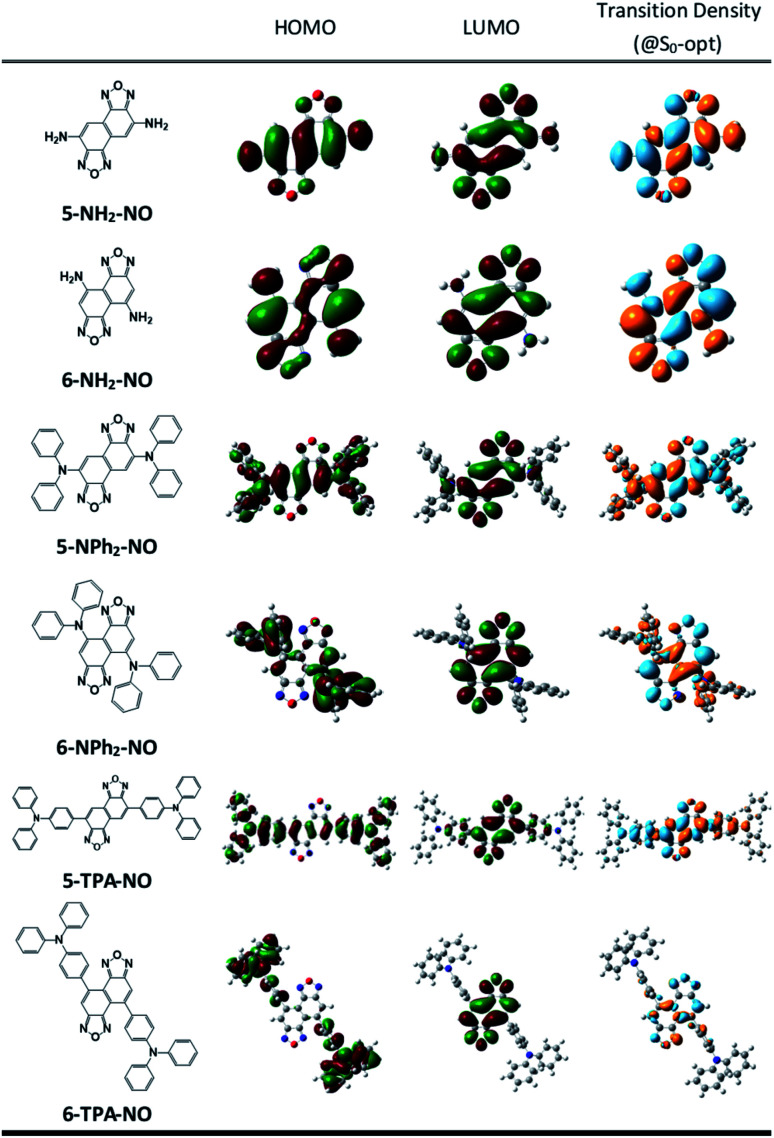
Chemical structures, frontier molecular orbitals and transition densities of D–A models: 5-NH_2_–NO, 6-NH_2_–NO (dashed lines represent nodes across the atoms. 6-NH_2_–NO exhibits two such nodes that are absent in 5-NH_2_–NO), 5-Ph_2_–NO, 6-Ph_2_–NO, 5-TPA–NO and 6-TPA–NO.

We then suspect that the donor–acceptor molecules may also exhibit subtle symmetry or substitution effects similar to the linear cyanine systems. Various donor–acceptor models are considered consisting of typical acceptors, NO (for X = O) or NT (for X = S) with donors ranging from simple amines, NH_2_ or NMe_2_, to more realistic NPh_2_ and triphenylamines (TPAs), substituted at the 5- or 6-position, represented as 5-D–A or 6-D–A, respectively (see Scheme 1). The computed optical excitations and detailed molecular orbital contributions are shown in Tables S4 and S5.† For systems with simple amine donors, D = NH_2_ and NMe_2_ the lowest excitation is dominated by the LE transition character, particularly for the 5-substituted systems (Fig. 4). Note that the reorganization energies of 6-NR_2_–NO (R = H or Me) systems are 1.5 times lower than those of the 5-NR_2_–NO counterparts (Table 2), indicating that there is indeed a symmetry or positional effect in the reorganization energy magnitude. The transition densities show two additional nodes across the atoms in 6-NH_2_–NO, represented by the dashed lines in Fig. 4, which are absent in 5-NH_2_–NO and the results are consistent with the results of linear cyanines (*vide supra*).

**Table 2:** Reorganization energy (kcal mole^−1^) and ratio of various D–A models^a^

D	A	*λ*_5-D–A_	*λ*_6-D–A_	*λ*_5-D–A_/*λ*_6-D–A_
NH_2_	NO	12.87	8.36	1.54
NMe_2_	NO	15.76	10.57	1.49
NPh_2_	NO	7.88	9.26	0.85
TPA	NO	10.56	10.78	0.98
TPA	NT	9.77	10.08	0.97

a See Scheme 1 for the structures of various D–A models.

In larger D–A models such as 5-NO–TPA and 5-NT–TPA, natural transition orbital (NTO) analyses reveal a majority of CT transitions from the hole located on TPA to the electron located on NO or NT, combined with a slight contribution of LE at the central core, whereas 6-NO–TPA and 6-NT–TPA present exclusively CT transitions (Fig. 4). However, it appears that for systems with almost pure CT type transitions there is no difference between 5- and 6-NT–TPA (Table 2). It seems that a certain extent of local excitation (LE) character is necessary for the substitutional position to be effective for possible reduction of the reorganization energy, as shown in 5(6)-NPh_2_–NO. When the LE character is increased in 5-NPh_2_–NO, the reorganization energy decreases compared with isomeric 6-NPh_2_–NO dominated by pure CT excitation, giving rise to a reversed *λ*_5-D–A_/*λ*_6-D–A_ of 0.85.

## Discussion

The above first principles approaches have led to a crucial finding that there exists a reflectional symmetry, characterized by the presence of a nodal plane of the transition density across the central carbon atom in the polymethine chain, plays a key role in significant reduction of the reorganization energy. Here, we attempt to translate this relationship into a more general chemical interpretation.

**Fig. 5:**
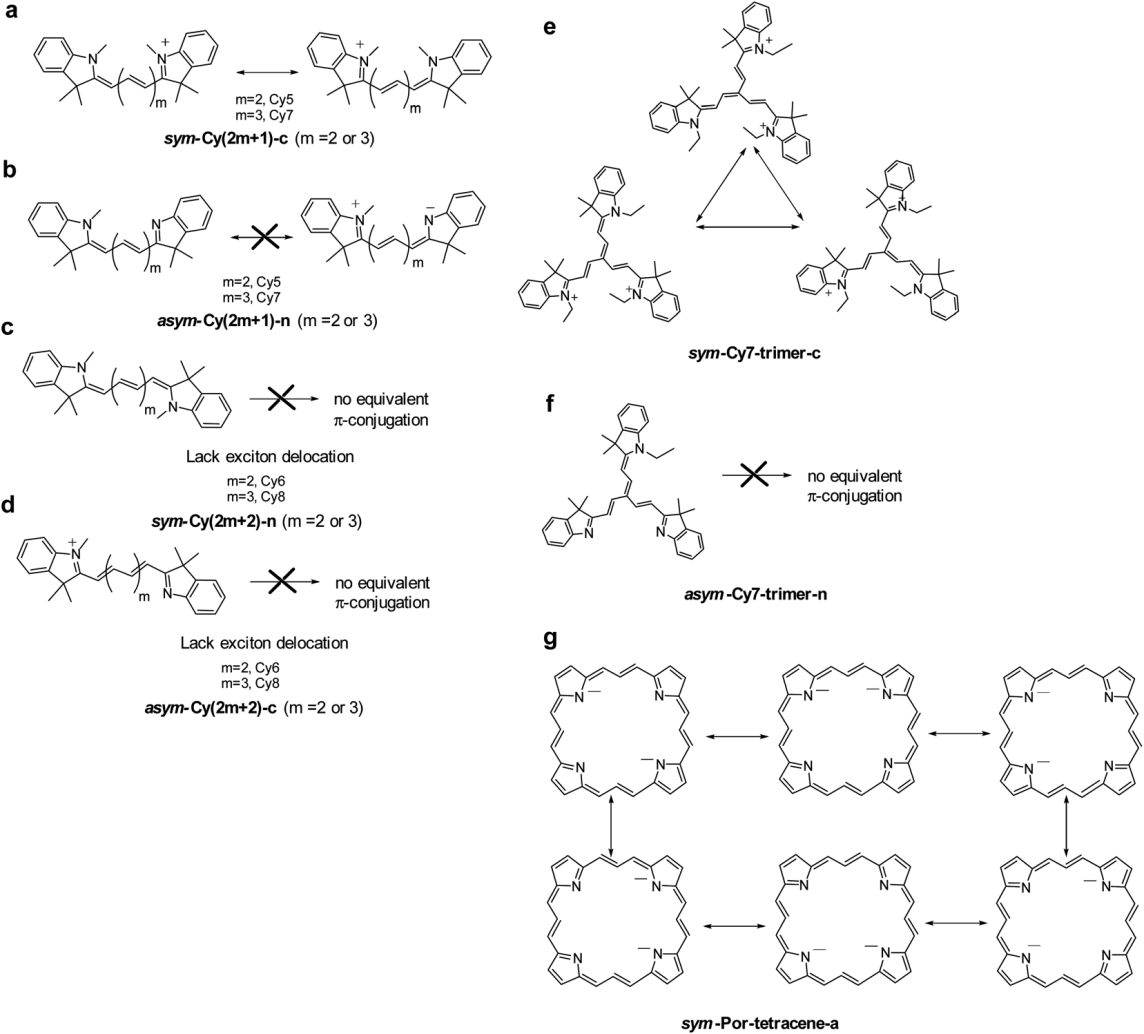
The canonical drawing of possible equivalent π-conjugations that lead to intramolecular exciton delocalization.

If one treats the lowest electronic transition of the title cyanines with a one-dimensional π-electron motion between two terminal nitrogen atoms using *e.g.*, the Hückel approximation, from a topological point of view, the transition characteristic depends on the type of π-conjugation and the atomic identity along the N-terminals. In other words, the exciton property can be described by canonically drawing π-conjugation incorporating alternative single-double bonds between two N-terminals. As shown in Fig. 5a, the π-electron configuration for both *sym*-Cy5-c and *sym*-Cy7-c can be depicted by the conjugation of the π electron terminated at either the left or right side of the cationic nitrogen. Upon excitation, these two electronic configurations should create a superposition of two identical exciton wavefunctions with equal probability.

Theoretically, the creation of a superposition of N exciton wavefunctions can be envisaged as N exciton delocalization modes. This viewpoint is similar to the consequence of exciton delocalization commonly encountered in semiconductor nanomaterials^92^ or self-assembled organic/inorganic packing such as square planar Pt(ii) complexes^93^ where the exciton can be largely delocalized. If one must tell the difference, we can plausibly consider the exciton delocalization in *sym*-Cy5-c and *sym*-Cy7-c as the intramolecular type as opposed to the intermolecular type in semiconductor quantum dots and self-assembled molecular solids. The *N* number of exciton delocalization then equally shares the reorganization energy *λ*, reducing the effective *λ*, *λ*_eff_, to *λ*/*N*. Classically, this can be imagined by the vibrational energy being equally dissipated to the *N* exciton delocalization modes. The reduced *λ*/*N* value should lead to a decrease of the non-radiative decay rate *k*_nr_ according to the energy gap law expressed in eqn (2). As depicted in Fig. 5a, *sym*-Cy5-c and *sym*-Cy7-c can be drawn by two resonating π-conjugations, effectively doubling the exciton delocalization. Therefore, *λ*_eff_ values for both *sym*-Cy5-c and *sym*-Cy7-c are predicted to be reduced by half with respect to their respective congeners that have solely one type of π-conjugation. The latter can be represented by *asym*-Cy5-n and *asym*-Cy7-n as shown in Scheme 1 and Fig. 5b, where two canonical π-conjugations are in neural and zwitterionic configurations and are obviously not equivalent. One thus predicts the reorganization energy of *sym*-Cy5-c and *sym*-Cy7-c to be more or less half of that in *asym*-Cy5-n and *asym*-Cy7-n. This prediction is consistent with the calculated values of 6.44 kcal mole^−1^ (*sym*-Cy5-c) and 5.86 kcal mol^−1^ (*sym*-Cy7-c), which are about half of 12.41 kcal mol^−1^ and 13.70 kcal mol^−1^ calculated for *asym*-Cy5-n and *asym*-Cy7-n, respectively (see Table 1). From the symmetry point of view, two equivalent excitons depicted along the forward and reverse linear directions shown in Fig. 5a are virtually the same as having a symmetry of the reflection plane, *i.e.* a nodal plane, at the central carbon atom of both *sym*-Cy5-c and *sym*-Cy7-c, supporting the conclusion made by the results of transition density.

When there are even numbers of carbon atoms, independent of symmetric or asymmetric systems, Cy6 or Cy8, simple trial and error exercises promptly reveal that no other possible equivalent π-configurations can be drawn (see Fig. 5c and d). This is also consistent with the lack of symmetry of the reflection plane concluded in the theoretical approach. As a result, the reorganization energy was calculated to be higher than 12 kcal mol^−1^ for all cyanines containing even numbers of carbon atoms (see Table 1).

Considering that a higher extent of exciton delocalization leads to a smaller reorganization energy, we also consider the possibility of *N* = 3, *i.e.*, a three-arm cyanine structure represented by a Cy7 trimer, classified as *sym*-Cy7-trimer-c and *asym*-Cy7-trimer-n (see Scheme 1). As for the *sym*-Cy7-trimer-c, shown in Fig. 5e, simple canonical drawing indicates that the π-conjugation can be equally distributed among the three ethyl-substituted branches. Nevertheless, this simple depiction of canonical triple π-resonance is largely prohibited by the deviation of planarity for the three cyanine arms in the *sym*-Cy7-trimer-c (*vide supra*) and can only occur at most between two of the three arms, similar to that depicted for *sym*-Cy7-c in Fig. 5a. Computationally, the reorganization energy for *sym*-Cy7-trimer-c was calculated to be 8.44 kcal mole^−1^, close to 5.80 kcal mole^−1^ calculated for *sym*-Cy7-c. The slightly larger value for *sym*-Cy7-trimer-c is believed to originate from the relatively non-planar configuration for the three cyanine arms in the *sym*-Cy7-trimer-c (see Table S3†).

On the other hand, the reorganization energy of *asym*-Cy7-trimer-n, lacking any other equivalent π-configuration, was calculated to be as large as 12.73 kcal mol^−1^ and is 1.5 times higher than that of *sym*-Cy7-trimer-c. Extending the concept to the 2D cyanine model, due to its six equivalent configurations shown in Fig. 5g, the porphyrin-like, *D*_4h_ symmetric structure possesses virtually zero reorganization energy compared with the corresponding asymmetric structure whose energy was calculated to be 6.71 kcal mole^−1^. Furthermore, an analysis of the bond length displacement reveals almost identical structures of *sym*-Por-tetracene-a in both S_0_ and S_1_ optimized structures. As for the D–A composites such as 5-D–A and 6-D–A shown in Scheme 1, if D is a strong electron donating group such as triphenyl amine where the HOMO and LUMO are separately dominated by the donor and acceptor, respectively (see Fig. 5), the resulting charge transfer excitation cannot be represented by π-electron delocalization. As a result, independent of donor substitution positions, the reorganization energy should be similar, supported by the calculated *λ*_inner_ of ∼10 kcal mol^−1^ for all designed 5-D–A or 6-D–A composites (where D = TPA and A = NO or NT). In fact, for the strong charge transfer character, it has been established that the outer-sphere reorganization energy, *e.g.* the polarity of solvent molecules in solution, plays an important role in the non-radiative decay rates. As for the weak electron donating groups such as –NH_2_ and NMe_2_, shown in Fig. 4, the delocalization of the π-configuration may start to show a positional effect for possible reduction of the reorganization energy. However, unlike the 1-D cyanine linear chain system, the π topology of such a 2D conjugated 5-NR_2_–NO or 6-NR_2_–NO (R = H and Me) system is complicated, and the determination of equivalent exciton delocalization by canonically drawing the π-conjugation becomes infeasible. Nevertheless, as elaborated early, the calculated 6-NH_2_–NO does possess additional nodal planes (*cf.* 5-NH_2_–NO) and thus has a lower reorganization energy than that of 5-NH_2_–NO.

## Conclusion

In summary, we have discussed the structural effect on the reorganization energy by comparing two major classes of molecules with subtle geometry adjustments. These two models consist of conjugated cyanine dyes and D–A composites with various degrees of CT character. For linear cyanine systems, we find that the cyanine molecule can have a drastic reduction in the reorganization energy if and only if it has a strict reflection symmetry, characterized by the presence of a nodal plane of the transition density across the central carbon atom in the polymethine chain. For donor–acceptor models with the NO or NT acceptor, a weak electron donor such as –NH_2_ with a local excitation character also exhibits a positional effect for possible reduction of the reorganization energy when additional nodes across the atoms are observed in the transition density. Nevertheless, this effect is absent in systems with strong charge transfer properties, *e.g.* using TPA as a donor. We also correlate the reflection of symmetry with the topological drawing of the number of resonating π-conjugations that represents exciton delocalization. As a result, the existence of the symmetry of reflection increases the extent of intramolecular type exciton delocalization, thus reducing the reorganization energy. Most notably, compared with reorganization energies of intermolecular type exciton delocalization, the energies of intramolecular type can be fine-tuned by molecular design rather than molecular aggregation, which provides a credible strategy to design organic dyes for near-infrared fluorescence imaging. The symmetry effect proposed in this study should be applicable to other systems with a lower emission energy gap in the NIR region to strategically improve the emission efficiency.

## Author contributions

C. C. W. conducted the DFT calculations and analysed the data. All the authors discussed the results and commented on the manuscript.

## Conflicts of interest

There are no conflicts to declare.

## Supplementary Material

SC-013-D2SC01851A-s001
