# Supplementary material for: Reducing the internal reorganization energy via symmetry controlled π-electron delocalization
Source: Chem Sci. 2022 May 24;13(24):7181–9. doi: 10.1039/d2sc01851a (PMC9214956; doi:10.1039/d2sc01851a)
Supplement: SC-013-D2SC01851A-s001 [file SC-013-D2SC01851A-s001.pdf]

Electronic Supplementary Material (ESI) for Chemical Science.
This journal is © The Royal Society of Chemistry 2022

Supporting Information

Reducing the Internal Reorganization Energy via Symmetry Controlled π-electron Delocalization

Chi-Chi Wu[a], Elise Y. Li[a*] and Pi-Tai Chou[b*]

[a] Department of Chemistry, National Taiwan Normal University, No. 88, Section 4, Tingchow Road, Taipei 116, Taiwan.

[b] Department of Chemistry, National Taiwan University, No. 1, Section 4, Roosevelt Road, Taipei 106, Taiwan.

Table S1. Computational results (and experimental values in parentheses) of absorption and emission wavelength (in nm) for cyanine systems with different functionals (a)ωB97XD (b)B3LYP

(a)

	Sym		*Asym*	
	Absorption	Emission	Absorption	Emission
**Cy5**	506.8 (652)	588.5(675)	409.8	574.8
**Cy6**	390.1	531.9	438.7	663.0
**Cy7**	590 (750)	694.7(775)	436.5	647.2
**Cy8**	416.0	598.0	466.3	712.3
**Cy7-trimer**	489.6	588.4	446.9	603.7
**Por-tetracene**	925.3	1189.6	668.4	1342.1

(b)

	Sym		*Asym*	
	Absorption	Emission	Absorption	Emission
**Cy5**	539.5 (652)	628.8(675)	490.8	600.8
**Cy6**	462.8	575.6	561.6	708.9
**Cy7**	601.4 (750)	699.4(775)	444.7	690.3
**Cy8**	507.6	646.9	603.2	766.4

Table S2. Optical excitation and molecular orbital contributions of cyanine models (a) symmetric and asymmetric linear cyanines (b) symmetric and asymmetric trimeric cyanines (c) symmetric and asymmetric porphyrin-6, 13, 19, 26-tetracene

(a)

		no.	E/eV	nm	f	Contribution	weight
***sym*-Cy5-c**	**Absorption**	S_1	2.45	506.8	2.1805	HOMO→LUMO	95%
	**(@S_0-opt)**	S_2	4.16	297.7	0.0423	HOMO-1→LUMO	82%
	**Emission**	S_1	2.11	588.5	2.3071	HOMO→LUMO	95%
	**(@S_1-opt)**	S_2	3.85	321.8	0.0166	HOMO-1→LUMO	81%
***asym*-Cy5-n**	**Absorption**	S_1	3.02	409.8	2.1639	HOMO→LUMO	91%
	**(@S_0-opt)**	S_2	4.40	281.4	0.1231	HOMO-1→LUMO	53%
						HOMO→LUMO+1	33%

	**Emission**	S_1	2.16	574.8	2.4457	HOMO→LUMO	95%
	**(@S_1-opt)**	S_2	3.90	317.7	0.0468	HOMO-1→LUMO	72%
***sym*-Cy6-n**	**Absorption (@S_0-opt)**	S_1	3.18	390.1	2.6178	HOMO→LUMO	93%
		S_2	4.28	289.7	0	HOMO-1→LUMO	28%
						HOMO-1→LUMO+2	16%
						HOMO→LUMO+1	38%
	**Emission (@S_1-opt)**	S_1	2.34	531.9	2.7151	HOMO→LUMO	96%
		S_2	3.96	312.6	0	HOMO-1→LUMO	54%
						HOMO→LUMO+1	17%
						HOMO→LUMO+3	17%
***asym*-Cy6-c**	**Absorption**	S_1	2.83	438.7	2.3539	HOMO→LUMO	85%
	**(@S_0-opt)**	S_2	3.96	313.4	0.0013	HOMO-1→LUMO	70%
	**Emission**	S_1	1.87	663.0	2.6419	HOMO→LUMO	94%
	**(@S_1-opt)**	S_2	3.26	379.8	0.0112	HOMO-1→LUMO	83%
***sym*-Cy7-c**	**Absorption**	S_1	2.08	594.8	2.1143	HOMO→LUMO	94%
	**(@S_0-opt)**	S_2	3.77	328.8	0.2644	HOMO-1→LUMO	80%
	**Emission**	S_1	1.75	708.2	2.2379	HOMO→LUMO	95%
	**(@S_1-opt)**	S_2	3.49	355.1	0.2632	HOMO-1→LUMO	77%
***asym*-Cy7-n**	**Absorption (@S_0-opt)**	S_1	2.80	443.3	2.0415	HOMO→LUMO	88%
		S_2	4.03	307.6	0.2734	HOMO-1→LUMO	68%
						HOMO→LUMO+1	20%
	**Emission (@S_1-opt)**	S_1	1.82	679.6	2.2335	HOMO→LUMO	95%
		S_2	3.44	359.9	0.3443	HOMO-1→LUMO	75%
						HOMO→LUMO+1	17%
***sym*-Cy8-n**	**Absorption**	S_1	2.98	416.0	3.1244	HOMO→LUMO	92%
	**(@S_0-opt)**	S_2	4.07	304.3	0	HOMO-1→LUMO	66%
	**Emission**	S_1	2.07	598.0	3.1792	HOMO→LUMO	96%
	**(@S_1-opt)**	S_2	3.63	341.8	0	HOMO-1→LUMO	72%
***asym*-Cy8-c**	**Absorption (@S_0-opt)**	S_1	2.66	466.3	2.8074	HOMO→LUMO	84%
		S_2	3.83	323.8	0.0258	HOMO-1→LUMO	57%
						HOMO→LUMO+1	25%
	**Emission (@S_1-opt)**	S_1	1.74	712.28	3.0994	HOMO→LUMO	93%
		S_2	3.13	396.53	0.0066	HOMO-1→LUMO	78%
						HOMO→LUMO+1	15%

(b)

		no.	E/eV	nm	f	Contribution	weight
***sym*-Cy7-trimer**	**Absorption**	S_1	2.53	489.6	1.3143	HOMO→LUMO	87%

	**(@S_0-opt)**	S_2	2.55	486.9	1.099	HOMO→LUMO+1	86%
	**Emission**	S_1	2.11	588.4	1.4742	HOMO→LUMO	94%
	**(@S_1-opt)**	S_2	2.60	477.5	1.0403	HOMO→LUMO+1	86%
***asym*-Cy7-trimer**	**Absorption**	S_1	2.77	446.9	1.2682	HOMO→LUMO	93%
	**(@S_0-opt)**	S_2	3.49	355.1	1.1234	HOMO→LUMO+1	78%
	**Emission**	S_1	2.05	603.7	1.2613	HOMO→LUMO	94%
	**(@S_1-opt)**	S_2	2.85	434.4	1.461	HOMO→LUMO+1	88%

(c)

		no.	E/eV	nm	f	Contribution	weight
***sym*-Por-tetracene**	**Absorption (@S_0-opt)**	S_1	1.34	925.27	0.0287	HOMO-1→LUMO+1	35%
						HOMO→LUMO	65%
		S_2	1.72	720.81	0.051	HOMO-1→LUMO	29%
						HOMO→LUMO+1	71%
	**Emission (@S_1-opt)**	S_1	1.04	1189.6	0.1290	HOMO-1→LUMO+1	28%
						HOMO→LUMO	80%
		S_2	1.09	1132.6	0.0127	HOMO-1→LUMO	43%
						HOMO→LUMO+1	66%
***asym*-Por-tetracene**	**Absorption (@S_0-opt)**	S_1	1.85	668.4	0.0341	HOMO-1→LUMO	37%
						HOMO→LUMO	60%
		S_2	2.44	509.0	0.1687	HOMO→LUMO	79%
	**Emission (@S_1-opt)**	S_1	0.92	1342.1	0.0403	HOMO-1→LUMO+1	42%
						HOMO→LUMO	74%
		S_2	1.28	971.2	0.0238	HOMO-1→LUMO	44%
						HOMO→LUMO+1	62%

Table S3. Dihedral angles (D1, D2 and D3) of *sym*-Cy7-trimer

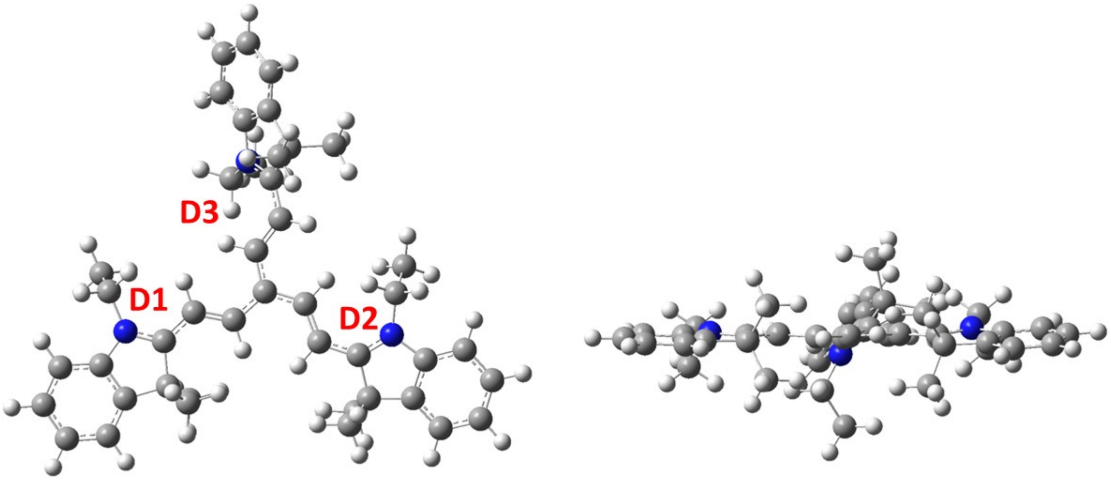

top view side view

	@S_0-opt	@S_1-opt
**D1**	179.11°	177.22°
**D2**	16.89°	10.24°
**D3**	-31.44°	-20.44°

Table S4. Computational (and Experimental) results of Absorption and Emission wavelength (in nm) in D-A compounds

D	A	5-D-A		6-D-A	
		Absorption	Emission	Absorption	Emission
NH_2	NO	452.6	631.3	417.8	504.7
NMe_2	NO	465.6	668.6	401.6	520.6
NPh_2	NO	437.6	544.3	491.6	643.0
TPA	NO	439.1 (542)	591.3 (603)	363.7	568.3

TPA	NT	453.8 (503)	561.2 (579)	368.4	471.1

Table S5. Optical excitation and molecular orbital contributions of donor-acceptor models, 5-D-A and 6-D-A (D=NH_2, NMe_2 and TPA) (a) A=NO(b) A=NT

(a)

		no.	E/eV	nm	f	Contribution	weight
**5-NH_2-NO**	**Absorption**	S_1	2.74	452.6	0.2503	HOMO→LUMO	98%
	**(@S_0-opt)**	S_2	3.63	341.9	0	HOMO→LUMO+1	94%
	**Emission**	S_1	1.96	631.3	0.2616	HOMO→LUMO	99%
	**(@S_1-opt)**	S_2	0.90	426.7	0	HOMO→LUMO+1	96%
**6-NH_2-NO**	**Absorption**	S_1	2.97	417.8	0.256	HOMO→LUMO	98%
	**(@S_0-opt)**	S_2	3.38	366.6	0	HOMO-1→LUMO	98%
	**Emission**	S_1	2.46	504.7	0.3287	HOMO→LUMO	98%
	**(@S_1-opt)**	S_2	3.00	412.5	0	HOMO-1→LUMO	98%
**5-NMe_2-NO**	**Absorption**	S_1	2.66	465.6	0.3359	HOMO→LUMO	97%
	**(@S_0-opt)**	S_2	3.56	348.6	0.0001	HOMO→LUMO+1	89%
	**Emission**	S_1	1.85	668.6	0.3209	HOMO→LUMO	98%
	**(@S_1-opt)**	S_2	2.75	450.2	0	HOMO→LUMO+1	96%
**6-NMe_2-NO**	**Absorption**	S_1	3.09	401.6	0.1778	HOMO→LUMO	98%
	**(@S_0-opt)**	S_2	3.34	370.6	0.0042	HOMO-1→LUMO	98%
	**Emission**	S_1	2.38	520.6	0.2385	HOMO→LUMO	98%
	**(@S_1-opt)**	S_2	2.77	446.9	0.0057	HOMO-1→LUMO	98%
**5-TPA-NO**	**Absorption**	S_1	2.82	439.0	1.3174	HOMO→LUMO	70%
	**(@S_0-opt)**	S_2	3.46	357.8	0	HOMO－1→LUMO	73%
	**Emission**	S_1	2.10	591.3	1.5035	HOMO→LUMO	87%
	**(@S_1-opt)**	S_2	3.10	399.4	0	HOMO－1→LUMO	73%
**6-TPA-NO**	**Absorption**	S_1	3.12	397.2	1.1699	HOMO-2→LUMO	19%
						HOMO→LUMO	74%
	**(@S_0-opt)**	S_2	3.83	323.7	0.0011	HOMO-1→LUMO	73%
	**Emission**	S_1	2.18	568.3	1.2155	HOMO→LUMO	90%

	**(@S_1-opt)**	**S_2**	3.42	362.2	0.0034	HOMO-1→LUMO	84%
**5-Nph_2-NO**	**Absorption (@S_0-opt)**	**S_1**	2.52	491.6	0.5679	HOMO→LUMO	93%
		**S_2**	3.38	366.4	0.00	HOMO-1→LUMO	44%
						HOMO→LUMO+1	51%
	**Emission (@S_1-opt)**	**S_1**	1.93	643.0	0.6554	HOMO→LUMO	95%
		**S_2**	2.99	415.2	0.00	HOMO-1→LUMO	31%
						HOMO→LUMO+1	64%
**6- Nph_2-NO**	**Absorption (@S_0-opt)**	**S_1**	2.83	437.6	0.00	HOMO→LUMO	92%
		**S_2**	2.88	430.8	0.0994	HOMO－1→LUMO	92%
	**Emission (@S_1-opt)**	**S_1**	2.28	544.3	0.0355	HOMO→LUMO	96%
		**S_2**	2.47	502.0	0.0538	HOMO－1→LUMO	96%

(b)

		**no.**	**E/eV**	**nm**	**f**	**Contribution**	**weight**
**5-NH_2-NT**	**Absorption**	**S_1**	2.67	464.8	0.1856	HOMO→LUMO	98%
	**(@S_0-opt)**	**S_2**	3.38	3671	0	HOMO→LUMO+1	95%
	**Emission**	**S_1**	1.89	656.6	0.2015	HOMO→LUMO	98%
	**(@S_1-opt)**	**S_2**	2.65	468.0	0	HOMO→LUMO+1	97%
**6-NH_2-NT**	**Absorption**	**S_1**	2.80	442.7	0.4195	HOMO→LUMO	97%
	**(@S_0-opt)**	**S_2**	3.32	372.8	0	HOMO-1→LUMO	96%
	**Emission**	**S_1**	2.34	528.6	0.5499	HOMO→LUMO	98%
	**(@S_1-opt)**	**S_2**	2.99	414.0	0	HOMO-1→LUMO	96%
**5-NMe_2-NT**	**Absorption (@S_0-opt)**	**S_1**	3.53	351.3	0.298	HOMO→LUMO	96%
		**S_2**	3.54	350.5	0.0012	HOMO-2→LUMO+1	17%
						HOMO-1→LUMO	76%
	**Emission**	**S_1**	1.84	671.9	0.2624	HOMO→LUMO	98%
	**(@S_1-opt)**	**S_2**	2.60	477.2	0	HOMO→LUMO+1	96%
**6-NMe_2-NT**	**Absorption**	**S_1**	2.89	428.2	0.3091	HOMO→LUMO	97%
	**(@S_0-opt)**	**S_2**	3.29	377.1	0.0061	HOMO-1→LUMO	97%
	**Emission**	**S_1**	2.28	542.7	0.4176	HOMO→LUMO	98%

	**(@S$_1$-opt)**	**S$_2$**	2.84	436.3	0.0097	HOMO-1→LUMO	97%
**5-TPA-NT**	**Absorption**	**S$_1$**	2.73	453.8	0.9992	HOMO→LUMO	84%
	**(@S$_0$-opt)**	**S$_2$**	3.20	387.2	0	HOMO-1→LUMO	88%
	**Emission**	**S$_1$**	2.21	561.2	1.1952	HOMO→LUMO	86%
	**(@S$_1$-opt)**	**S$_2$**	3.17	391.1	0	HOMO-1→LUMO	57%
						HOMO→LUMO+1	28%
**6-TPA-NT**	**Absorption**	**S$_1$**	3.36	368.4	0.1698	HOMO-2→LUMO	38%
						HOMO→LUMO	57%
	**(@S$_0$-opt)**	**S$_2$**	3.57	346.8	0.0018	HOMO-1→LUMO	84%
	**Emission**	**S$_1$**	2.63	471.1	0.2472	HOMO→LUMO	82%
	**(@S$_1$-opt)**	**S$_2$**	3.26	380.6	0.1115	HOMO-2→LUMO	33%
						HOMO-1→LUMO	48%

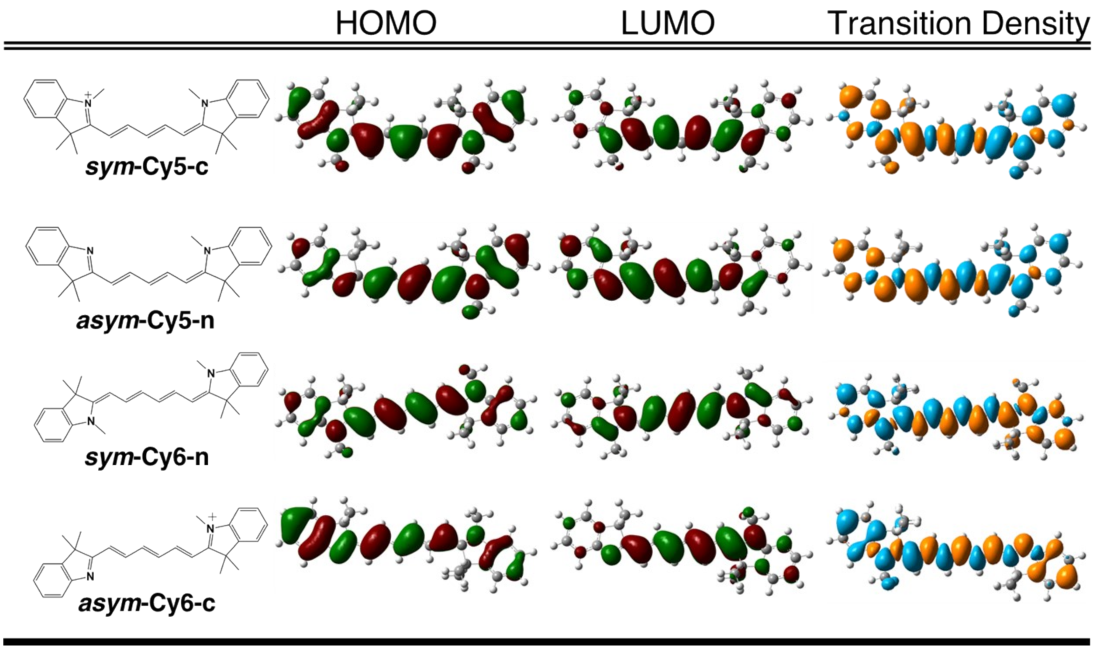

Figure S1. Frontier molecular orbitals and transition density of symmetric and asymmetric cyanine systems (m=2)

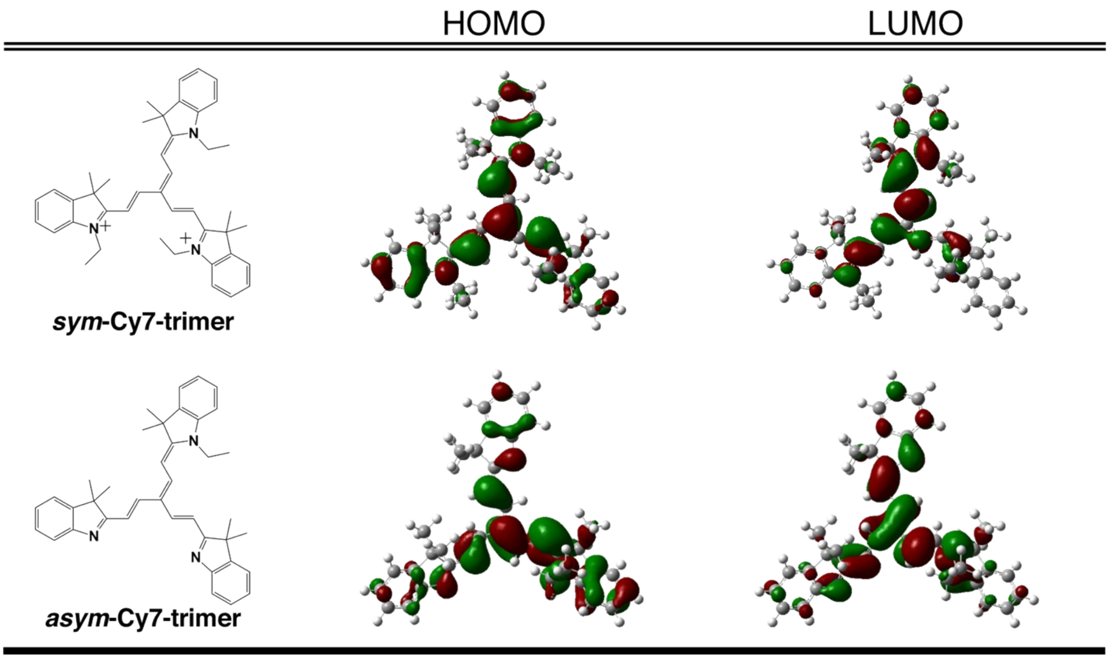

Figure S2. Frontier molecular orbitals of symmetric and asymmetric Cy7-trimer

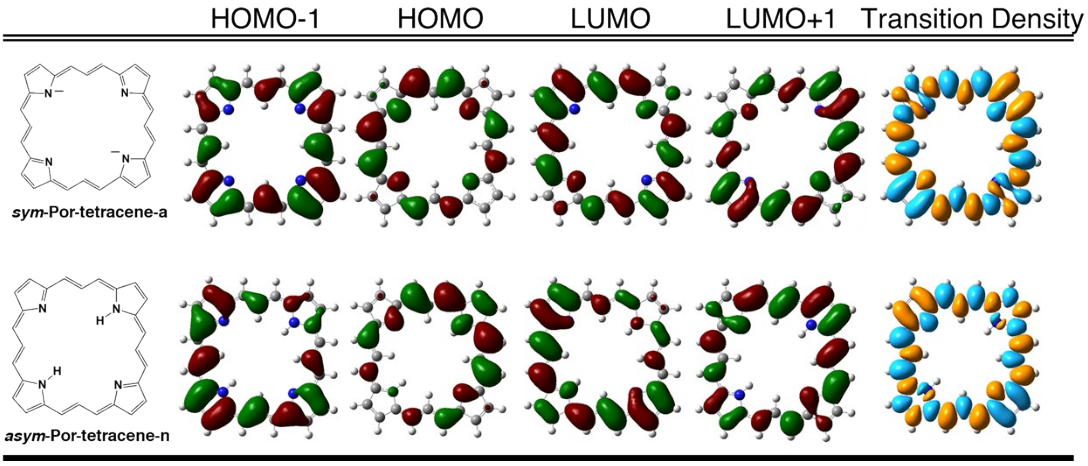

Figure S3. Frontier molecular orbitals and transition density of symmetric and asymmetric Por-tetracene

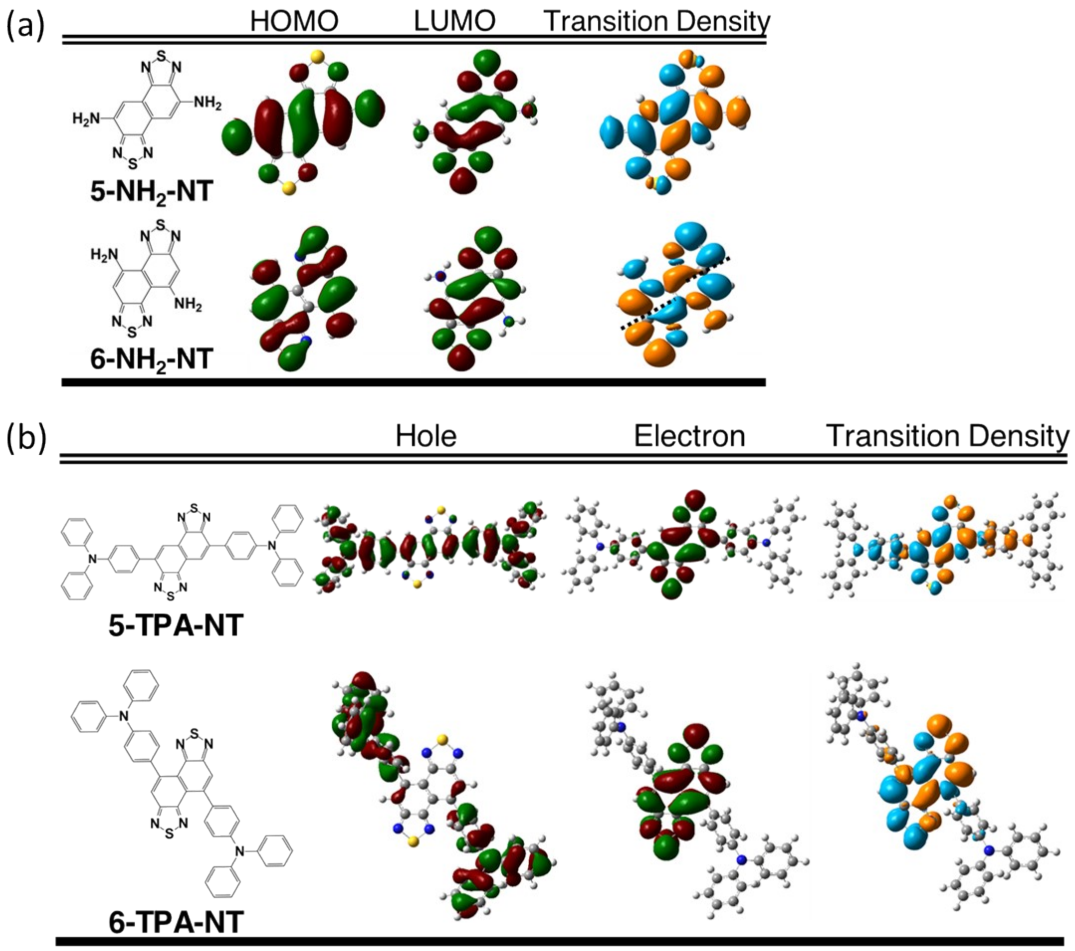

Figure S4. NTO analysis and transition density of (a) 5-NMe_2-NT and NMe_2-NT (b) 5-TPA-NT and 6-TPA-NT
